# The evolution of socioeconomic health inequalities in Ecuador during a public health system reform (2006–2014)

**DOI:** 10.1186/s12939-018-0905-y

**Published:** 2019-02-08

**Authors:** María Luisa Granda, Wilson Giovanni Jimenez

**Affiliations:** 1 Escuela Superior Politécnica del Litoral, ESPOL, Escuela de Postgrados en Administración de Empresas ESPAE, Campus Las Peñas, Malecón 100 y Loja, Guayaquil, Ecuador; 2 Área de postgrados en gestión de la salud y la seguridad social, Universidad Jorge Tadeo Lozano, Carrera 4 #, 22-61 Bogotá D.C., Colombia

**Keywords:** Health policy, Health inequality, Public health care services, Concentration index, Equity in utilization, Ecuador

## Abstract

**Background:**

One of the challenges in international health care policy is the reduction of health inequalities. We study the case of a recent health care reform in Ecuador, based on a revised model of public provision and financing of comprehensive care. The policy challenged not only the health care system but also key determinants of health, seeking to grant access and reduce persistent inequalities in utilization of health care services. This study evaluates the progress made on the socioeconomic health inequalities before and after reforms, between 2006 and 2014.

**Methods:**

The Living Standards Measurement Survey 2006 and 2014 are used to examine trends in inequality of two health dimensions: health status and health care utilization in Ecuador. The analysis includes the estimation and decomposition of the concentration index of health care utilization variables to understand which the most important contributors to inequality are, and how they change during the period analyzed.

**Results:**

Health inequality reduced after the public health system reform in Ecuador. The utilization of curative visits shows a pro-rich bias, which significantly reduced over the period of analysis. The use of a public facility for a doctor visit also shows a moderate reduction in socioeconomic inequality; although concentrated among the poor, the findings confirm an increase in the utilization of public health facilities by all socioeconomic groups. The decomposition analysis concludes that the most relevant determinants of the inequality in health care utilization variables are income, family size and education. Moreover, the contribution of income decreased dramatically in the utilization of curative visits after the reforms.

**Conclusion:**

This is the first study that assesses the inequality implications of recent policies targeted at guaranteeing the right to health, equity and social protection of the Ecuadorian population. We provide evidence of a significant reduction of health care inequality, following a well-rounded set of public interventions and investments to attack health and inequality determinants. Yet policies aimed at improving the distribution of education and income are necessary to further reduce health inequalities.

## Background

There is an established relationship, in literature, between socioeconomic conditions and health [1, 2]. Stiglitz [3] argues that income and wealth inequality affect health and reduce the life expectancy of the population. In recent years, an increasing inequality in the distribution of income and wealth has been observed, worsening living conditions for the population in low and middle-income groups, whereas 1% of the population concentrates 40% of wealth [4]. These figures have raised the global concern and efforts to understand not only income and wealth inequality, but also health inequality around the world.

Equity in health is defined as “the absence of systematic differences between populations or population subgroups defined socially, demographically or geographically, in one or more aspects of health” [5]. Health equity usually refers to the study of differences in dimensions such as health outcomes, health care utilization, subsidies and health care payments. This study explores variables in the first two dimensions.

In Latin America, the Pan American Health Organization (PAHO) has devoted resources and research efforts to explore the evolution of health inequalities. In 1998, a joint initiative between PAHO, the World Bank and UNDP supported a series of studies on investment in health, equity, and poverty reduction, named EquiLAC [6]. A few years later, a second round EquiLAC II [7] was launched to measure and explain health systems inequalities in six countries of the Americas: Brazil, Chile, Colombia, Jamaica, Mexico and Peru. Most of these countries had implemented policies, developed targeted programs and made legal reforms to increase coverage and access to health care, for the most vulnerable population, leading to improved equity in health outcomes and health care utilization [8].

The EquiLac Project included the study of the case of Ecuador [9], providing evidence that health expenditure and the use of health care services mostly benefited the population in higher-income groups using data from a household survey in 1995.

Other studies for Ecuador include Lopez-Cevallos & Chi [10] and Sanhueza et al. [11] providing understanding for socioeconomic determinants of health care use in Ecuador. They find strong evidence of inequalities in the Ecuadorian health care system, although Sanhueza et al. [11] focuses on maternal mortality inequalities and emphasizes on the need to understand the social determinants that contribute to health inequalities at a micro level. They agree on the need of a reform to the Ecuadorian health system to expand coverage, specifically to indigenous, low income and rural households. Our study considers the evolution of these findings and adds to them by using more recent data, before and after a period of reform in the line of previous literature recommendations.

### Historical context of the ecuadorian health system

The Ecuadorian health system has been under constant change since 1996 due to structural transformations in the economic and development model. The latest two constitutions, enacted in 1998 and 2008, have emerged from structural transformation processes that took place in Ecuador.

At the end of 1996, Ecuador began a period of democratic instability that lasted 10 years, with consequences in the performance of the health care system such as a reduction of the budget for health service provision, infrastructure worsening due to lack of investment, low quality of health care services and absence of a coherent institutional structure, all of them leading to increased out-of-pocket health care expenditures that affected all the health system users, especially the poor population [12, 13]. Within this context, the 1998 Constitution was approved, committing the State to guarantee the right to health, according to the principles of equity, universality, solidarity, quality and efficiency.

In 2001, a new Social Security Law was issued establishing general regulations for social security in Ecuador, regarding guiding principles, participants, risks covered and resources. It defined the institutions that were part of National Social Security System: Social Security Ecuadorian Institute (IESS for its Spanish acronym), Armed Forces Social Security Institute (ISSFA for its Spanish acronym), and the National Police Social Security Institute (ISSPOL for its Spanish acronym) [14]. IESS provided coverage for employee contributors (with partial contribution from employers), voluntary contributors and farmers or rural area residents. The coverage included health care benefits in the IESS network, a pension account, and access to mortgages and unsecured loans.

However, despite different government plans and strategies, by 2006, health and illness indicators showed scarce progress, partly due to political instability and the dollarization of the economy which affected living conditions of Ecuadorian families [13]. In 2006, the National Congress issued the Organic Health Law, driven by the need to update regulatory concepts in public health and human rights [15]. In the same year, the Inter-American Development Bank approved a loan for US 90 million dollars to support the project of Universal Health Insurance in Ecuador (PRO-AUS, for its Spanish acronym). The main objectives of the project were to give coverage to the poorest population in the country and to improve health services quality [16].

In 2008, the current Constitution was promulgated. It introduced a change in the right to health concept, as it was not only limited to the welfare of mind and body but included other areas related to public policy such as access to public services (water, sanitation, electricity), education, work, healthy environments and other services that guarantee or improve life quality and conditions of the population [13, 14].

This policy, along with the Social Development Agenda and the Millennium Development Goals, was key to build the Model of Comprehensive Family, Community and Intercultural Health Care (MAIS-FCI for its Spanish acronym). This model seeks to guarantee the right to health and social protection of the Ecuadorian population through a reform in the National Health System [13, 17].

In recent years, Ecuador stands out in the Latin American region for its sharp reduction in poverty, which went from 37.6% in 2006 to 22.5% in 2014, as well as its reduction in inequality with a Gini index of 53.2 in 2006 and 45.4 in 2014 [18, 19]. Starting in 2007, a public health reform was implemented to reduce financial and social barriers to health care, to strengthen public health care services and to improve their distribution.

The reforms were supported by an increasing public investment in health, specifically in hospital infrastructure, institutional restructuring, and the implementation of MAIS – FCI [12, 20]. According to the Global Health Observatory of World Health Organization [21], Ecuador’s total expenditure on health as a percentage of its GDP increased from 5.86 in 2006 to 9.16 in 2014, mainly driven by the growth of social security and government health expenditure, shown in Table 1.

**Table 1:** Health indicators in Ecuador (2006 & 2014)

Variable	2006	2014
Total expenditure on health as a percentage of GDP	5.86	9.16
Government expenditure on health as a percentage of total expenditure on health	24.31	49.21
Government expenditure on health as a percentage of total government expenditure	5.07	10.23
Social security expenditure on health as a percentage of government expenditure on health	23.41	50.24
Out of Pocket expenditure per capita in US$	116.5	230.3
Total number of Health care Facilities	3681	4139
MPH Facilities	1737	2099
IESS Facilities	51	74
Rural IESS Facilities	577	657
Ratio of Doctors (per 1000 population)	0.903	2.035
Ratio of Nurses (per 1000 population)	0.537	1.014
Under 5 mortality rate	28.3	21.5
Adult mortality rate	144	119
Prevalence of obesity among adults	14.6	18.3

Sources: WHO’s Global Health Observatory Data Repository [21], INEC [22]

The reforms pursued to increase access through the national health system, providing free of charge health care services to every citizen through the facilities of Ministry of Public Health (MPH). According to INEC [22] and presented in Table 1, the total number of health facilities in the country increased by 12% from 2006 to 2014, particularly driven by the expansion of the public network, MPH, IESS, and Rural IESS, and by the rise of private providers hired by public entities, specially IESS, to close the gap in demand for their health care services. The number of health professionals, specifically medical and nurse, doubled from 2006 to 2014. Part of the health surveillance and control policy, national strategies of immunization, HIV/AIDS, TB and maternal mortality were implemented. In the relevant period, adult mortality rate declined from 144 to 119 (probability of dying between 15 and 60 years per 1000 population), according to WHO’s Global Health Observatory.

Also, the Ecuadorian population gained access to public education, and improved sanitation, road infrastructure and labor conditions. Specifically, Constitutional Mandate 8 was established in 2008, to fight precarious labor conditions. It enforced worker’s mandatory enrollment of employees and benefits regulation, which include health care through IESS, with a separate provision network that was also strengthened as shown in Table 1.

As described above, during the period 2006–2014 the Ecuadorian health system expanded its capacity to increase the supply of public health care services and affect certain outcomes. Moreover, at a macroeconomic level, these were years of economic expansion: between 2008 and 2014, Ecuador experienced average annual increases of 4.6% in gross domestic product (GDP) [23]. However, how these developments have affected different socioeconomic groups, and hence, health equity in the country is yet unclear. This research seeks to provide some insights in this matter.

Specifically, this research intends to answer two questions: 1) How have socioeconomic inequalities in health status and health care utilization in Ecuador evolved between 2006 and 2014, following the enhancement of the role of the State in the health care system? 2) Which are the main determinants of socioeconomic health inequalities and how have they changed in the analyzed period? The key efforts in health policy described here made it necessary to measure and understand how health inequalities have evolved. The paper is structured as follows; the next section discusses the data and methodology applied to obtain estimates for the inequality in health and health care measures in Ecuador; section III explains the results; finally, a discussion and conclusions on the research are presented.

## Data and methodology

The Data source is the Living Standards Measurement Survey (LSMS) for the years 2006 and 2014 conducted in Ecuador by the National Institute of Statistics and Census (INEC). A full description of the survey is available at INEC [24]. This is a household survey representative at the country, region, and province levels, with urban and rural coverage. It contains specific information on key variables associated to household living conditions, such as housing, health, education, economic activity, migration, and household expenditures. The sampling design was probabilistic, stratified and proportional to population size; hence, the results are generalizable at the population level. Sampling weights are provided in the dataset. The sample size was computed to maintain a representative survey, adjusting it by non-response rates based on previous surveys.

We considered including in the analysis the previous rounds of the survey, dating back to 1998 and 1999. However, for the 1999 survey, the sample was not representative of the Ecuadorian population, since the Amazon region was not included for the survey. Additionally, 1999 was a period of severe economic and hyper-inflation crisis; even in 1998 some of the households started facing the burden of the crisis. In early 2000, the government decided to abandon the domestic currency to adopt the US dollar. All these issues, added to the difficulty to obtain comparable measures for the income variable, moved us to limit the analysis to 2006 and 2014, two separate time points which allow the study of trends in health inequality.

We decided to perform the analysis with adult population in the survey as is typically used in studies apart from children population, due to the inherent attributes of each sub-group in regard to health and health care needs.

### Measures

O’Donnell et al. [25] present a wide compendium of concepts, methods and examples in the study of health inequality. They categorize health outcomes, health care utilization, subsidies received and health care payments as the four key groups of focal variables in health inequality studies. This research focuses on the first two groups, health status and health care utilization to measure socioeconomic health inequality in Ecuador in 2006 and 2014. Health status is usually self-reported or assessed through different health indicators that may also differ according to the subject of study. Health care utilization is the outcome of the interaction between health professionals and patients. It can be assessed from the professional or the patients’ perspective. Patients’perspective although subjective, is available at a bigger scale through household and health surveys. Professionals’ perspective is usually more objective but requires the use of administrative records and good information systems [26]. We focus on adult population health status and health care services utilization from the patients perspective, in a similar way to that used in PAHO studies on Latin American countries.

Finally, to respond to the second objetive we use the decomposition approach to understand which are the key determinants in health care utilization inequalities and how they changed over time. The dependent variables in the analysis are grouped in the health status and health care utilization dimensions. We also group socioeconomic status, and control variables (need and non-need). Table 2 shows the definition of the variables and the transformations applied when necessary.

**Table 2:** Definition of variables

Dependent variables	Description
Health status	
Self-reported illness or accident	Categorical: In the past month, did you have an injury or illness that required medical attention? 1: Yes, 0: No
Health care utilization	
Any curative visit	Categorical: Due to the illness or injury, did you visit a doctor, nurse or “*curandero*”? 1: Yes, 0: No
Public facility visit	Categorical: Where did you go for a doctor visit the last time you required it? Public, Private. IESS and MPH are categorized as public doctor visits
Socioeconomic status	Description
Income	Adult equivalent per-capita income in constant US dollars. Includes labor income, income from capital gains, transfers, pensions, and other alimonies
Need control variables	Description
Age	Categorical. Five categories: 18–34, 35–44, 45–64, 65–74, 75- years.
Sex	Categorical. Reported in the survey: Male, Female
Restricted activity days	Numeric count: How many days did you stop doing your normal activities due to health problems? Number of days
Chronic condition	Categorical: Did your illness last over a year? 1: Yes, 0: No
Non-need control variables	Description
Education	Continuous. Years of schooling.
Employment status	Categorical. Employment status. Four categories: Employed, Underemployed, Unemployed, Economically inactive
IESS affiliation	Categorical: Are you affiliated or covered by any type of IESS (*general*, *voluntary*, *rural*, ISSFA o ISSPOL)? 1: Yes, 0: No
Marital status	Categorical: Reported in the survey. Married or civil union, Other (single, widow, separated, divorced)
Region	Categorical: Geographic region of the country. Three categories: Coastal Region; Highlands, Amazon
Rural	Categorical. 1: Yes, 0: No (Urban)
Indigenous	Categorical. Self-reported in the survey 1: Yes (Indigenous), 0: No (Other)
Household size	Number of people living in the household unit

### Data analysis

The methodology to be applied to study inequalities in the Ecuadorian population health status and health care utilization variables follows O’Donnell et al. [25] and Almeida & Sarti [8]. The main objective of the methodology is to measure and explain the evolution of inequalities, related to a socioeconomic status variable.

In order to measure health inequality, we compute the Concentration Index and the Horizontal Inequity Index. The first step requires the standardization of the health variable. We use the indirect method, which is more suitable when using micro-data. First, we estimate a regression of the form:

1 $$ {y}_i=\alpha +\sum \limits_j{\beta}_j{x}_{ji}+\sum \limits_k{\gamma}_k{z}_{ki}+{\varepsilon}_i $$

where

y_i_ is the health variable,

x_ji_ includes age, sex and health need variables, and

z_ki_ are the non-need variables.

The estimates of the coefficients allow us to compute the expected health for each individual

2 $$ {\widehat{y}}_i^X=\widehat{\alpha}+\sum \limits_j{\widehat{\beta}}_j{x}_{ji}+\sum \limits_k{\widehat{\gamma}}_k{\overline{z}}_k. $$

where ${\overline{z}}_k$ is the mean value of the zs.

Then, we generate the standardized health measure. The distribution of the standardized y is the distribution of health expected to be observed, irrespective of differences in the distribution of x’s related to income.

3 $$ {\widehat{y}}_i^{IS}={y}_i-{\widehat{y}}_i^X+\overline{y}. $$

The superscript IS indicates Indirect Standardization.

In this study, the health status variable is standardized by age and sex, while health care utilization variables are standardized by need (age, sex, number of days of restricted activity and the presence of a chronic condition) and non-need variables.

To implement the inequality analysis, we use the concentration index, a value that quantifies the degree of socioeconomic inequality in a health variable. It corresponds to twice the area between the concentration curve and the line of equality (45-degree line), and it is computed as described below using the convenient covariance formula for weighted data [8, 25]. (Eq. 4)

4 $$ CI=\frac{2}{\mu } Cov\left({y}_i,{r}_i\right) $$

where μ is the weighted sample mean of y, and r is the fractional rank in the socioeconomic standards distribution of the i-th individual, computed as

5 $$ {r}_i=\frac{1}{n}{\sum}_{j=1}^{i-1}{w}_j+\frac{1}{2}{w}_i $$

where n is the sample size and w are the sample weights. As the value of CI approaches zero, it represents absence of inequality.

In this study, all of the outcome variables are binary. The literature debate on the alternative indices for binary variables is vast; there are advantages and disadvantages for each index (see in the literature Kjellsson & Gerdtham [27] for an overview). We compute the inequality index CI (Eq. 4) to stay in the line of EquiLAC II analysis, since comparisons between contexts are affected by the choice of index.

There is a concern about the use of health status and health care utilization as dependent variables due to their binary nature, and of the appropriateness of linear models for the analysis. Almeida & Sarti [8] provide evidence of similar results using linear and non-linear models and argue on the advantages of this choice. We compute and compare the linear and non-linear approximations (probit) and decide to apply linear approximations for all estimations, as there are no significant differences in the results.

Next, the horizontal inequity index (HI) is computed. It measures the level of health inequality related to income, after differences in health needs across the income distribution are accounted for. It is calculated as the difference between the concentration index of the unstandardized health variable and the need-predicted distribution. That is,

6 $$ HI= CI- inequality\  due\  to\ need\ factors $$

For the second goal of this research, the assessment of the determinants of health inequality and its changes, the CI of the health care utilization variables is decomposed into the contributions of individual factors to income related health inequality as shown in Wagstaff et al. [28]. The contribution is computed as the product of the sensitivity of health with respect to that factor and the degree of income-related inequality in that factor.

For any linear additive regression model of health (y), such as

7 $$ y=\alpha +{\sum}_k{\beta}_k{x}_k+\varepsilon $$

where

x_k_ is the k-th health inequality determinant or factor (need and non-need)

and *ε* is the error term

The concentration index is defined as

8 $$ C={\sum}_k\left({\beta}_k\frac{{\overline{x}}_k}{\mu}\right){C}_k+{GC}_{\varepsilon }/\mu $$

where μ is the mean of y, ${\overline{x}}_k$ is the mean of x_k_, C_k_ is the concentration index for X_k_, and GCε is the generalized concentration index for the error term (ε). C is equal to a weighted sum of the concentration indices of the k regressors, where the weight for ${\overline{x}}_k$ is the elasticity of y with respect to ${\overline{x}}_k$. The residual should approach zero for a well specified model.

We describe the changes across time in the variables of interest, compute concentration indices and perform the decomposition of inequality indices by the different characteristics that according to the conceptual framework contribute to the measure. In this study, need variables are proxied by measures of expected health care utilization such as demographics (nine categories of interactions of age and sex, the excluded is male in the youngest group), the number of days of restricted activity due to health problems and the presence of a chronic condition. The non-need factors are the log of adult equivalent per-capita income, and individual characteristics such as education (in years of schooling), rural residence (located outside towns and cities, and in rural parishes), IESS affiliation, marital status, indigenous background, region of residence, employment status (three categories, except under-employment that is the reference category) and family size.

All the data analysis is performed with the Stata 14 software. In the next section, the results on the inequality measures of the performance of the health system in Ecuador, and its changes between 2006 and 2014 are presented.

## Results

Table 3 contains the descriptive statistics of the relevant sample in 2006 and 2014. The sample size is reported at the bottom of the table. We use sample weights to obtain all the results in the study.

**Table 3:** Descriptive statistics

	2006		2014	
Dependent variables				
Health status				
Self-reported illness or accident	0.55	(0.005)	0.48	(0.005)
Health care utilization				
Any curative visit	0.39	(0.005)	0.49	(0.006)
Public facility visit	0.40	(0.009)	0.62	(0.006)
Socioeconomic status				
Income	345.77	(10.144)	426.72	(9.319)
Need control variables				
Age				
18–34	0.44	(0.004)	0.41	(0.004)
35–44	0.20	(0.003)	0.20	(0.002)
45–64	0.25	(0.003)	0.27	(0.003)
65–74	0.06	(0.002)	0.07	(0.002)
75 -	0.05	(0.002)	0.05	(0.001)
Sex	0.48	(0.002)	0.48	(0.002)
Restricted activity days	7.79	(0.169)	7.37	(0.160)
Chronic condition	0.21	(0.008)	0.29	(0.009)
Non-need control variables				
Education	8.37	(0.092)	9.16	(0.077)
Employment status				
Employed	0.58	(0.005)	0.51	(0.003)
Underemployed	0.17	(0.004)	0.21	(0.004)
Unemployed	0.03	(0.001)	0.03	(0.001)
Economically inactive	0.22	(0.003)	0.25	(0.003)
IESS affiliation	0.21	(0.006)	0.39	(0.005)
Marital status	0.64	(0.004)	0.62	(0.004)
Region				
Coastal region	0.46	(0.013)	0.50	(0.007)
Highlands region	0.50	(0.014)	0.46	(0.006)
Amazon region	0.04	(0.001)	0.04	(0.001)
Rural	0.34	(0.013)	0.31	(0.009)
Indigenous	0.07	(0.006)	0.07	(0.003)
Household size	4.72	(0.035)	4.25	(0.021)
Sample size	32,469		66,418	

The variables are measured in decimal points, except for Income (real US dollars), restricted activity days (days), education (years of schooling) and household size (number of persons in the household). Standard errors are in parenthesis

A decline in the prevalence of self-reported illness contrasts with the growth in the share of people who attended curative visits and went to a public facility for a doctor visit, that grew over 50% between 2006 and 2014. Changes in inequality of these variables are discussed later.

The increase in per-capita income could be explained by a systematic increase in the minimum wage, implemented as a government policy starting on 2007. The monthly minimum wage in Ecuador increased from $170 in 2007 to $366 in 2016 (nominal terms) corresponding to 48% in real terms. This policy was accompanied by an enforcement of the law requiring employers to provide social security affiliation to their employees. Hence, this variable shows an increase of 0.18 between 2006 and 2014. In contrast, the proportion of people employed shows a reduction from 0.58 to 0.51, switching to underemployment or inactivity.

These changes in labor market indicators and other population characteristics, such as the average level of schooling, have influenced health inequality as discussed in the next sections.

### Standardized quintile distributions of health and health care utilization variables

The following table shows the standardized quintile distributions for the health status variable. The average probability of any illness or accident reduced from 0.55 in 2006 to 0.48 in 2014. A similar trend is observed in the quintiles, as the probability of illness decreases in time. The behavior of this variable provides an insight that health may have improved during the period of analysis; with a relative gain in health of the poorest greater than that of the richest. It is worth noticing that the lowest income quintile reached similar levels of self-reported illness in 2014 (0.51) when compared to the top income quintile in the baseline of the analysis (0.49) (Table 4).

**Table 4:** Standardized quintile distributions of health status and health care utilization variables (2006 and 2014)

	Year	Mean	Q1	Q2	Q3	Q4	Q5
Health status							
Self-reported Illness	2006	0.55	0.60	0.59	0.57	0.55	0.49
	2014	0.48	0.51	0.52	0.50	0.47	0.44
	Dif	−0.07^*^	−0.09^*^	−0.07^*^	−0.07^*^	−0.08^*^	−0.05^*^
Health care utilization							
Any curative visit	2006	0.37	0.25	0.35	0.35	0.38	0.53
	2014	0.48	0.39	0.44	0.49	0.51	0.56
	Dif	0.11^*^	0.14^*^	0.09^*^	0.14^*^	0.13^*^	0.03^*^
Public facility visit	2006	0.41	0.49	0.48	0.44	0.37	0.29
	2014	0.63	0.76	0.74	0.66	0.59	0.51
	Dif	0.22^*^	0.27^*^	0.26^*^	0.22^*^	0.22^*^	0.22^*^

The health status variable was standardized by demographics (age and sex). Health care utilization variables were standardized by need and non-need variables in Table 2. Statistical significance of the difference between the two periods is tested with mean comparison t-test for unpaired data ^*^ *p* < 0.01

Regarding health care utilization, the probability of a curative visit showed an increase from 2006 to 2014, at the average level and by quintiles; at higher income levels, the higher use of curative visits. The biggest changes occurred in quintiles 1 and 3, where the value increased in 0.14 providing evidence of increased utilization of health care services in these income groups.

The likelihood of using a public facility for a doctor visit has also increased in time for all income quintiles. We interpret the mean variation of 0.22 as evidence of increased utilization of the public health system since the change is substantial. Moreover, for groups that already had a high utilization level in 2006, Q1 and Q2, the variation was even bigger, 0.27 and 0.26 respectively.

### Concentration indices and horizontal inequity indices for health status and health care utilization variables

The degree of socioeconomic inequalities in health status and health care utilization variables was computed by using the concentration index and the horizontal inequity indices in the years 2006 and 2014. The concentration index (CI) for the unstandardized distribution of the variables and the horizontal inequity index (HII) are shown in Table 5. This allowed to appreciate the evolution of the index in time and across different measures of health status and health care utilization.

**Table 5:** Concentration indices and horizontal inequity indices for health status and health care utilization variables

Variable	2006		2014		Dif. 2014–2006	
	CI	HII	CI	HII	CI	HII
Self-reported illness	−0.056^*^	− 0.043^*^	−0.065^*^	− 0.047^*^	−0.009^*^	− 0.004
Any curative visit	0.058 ^*^	0.066 ^*^	0.015 ^*^	0.023 ^*^	−0.043 ^*^	−0.043 ^*^
Public facility visit	−0.102 ^*^	−0.100 ^*^	− 0.084 ^*^	−0.083 ^*^	0.018 ^*^	0.017 ^*^

*CI* Concentration index, *HII* horizontal inequity index

^*^P < 0.01

The self-reported presence of an illness or accident intends to approximate the health status of the individual. A negative value of the CI indicates that illness is concentrated among the poor, an empirical fact consistent with worldwide evidence [25]. According to the previous quintile analysis, self-reported illness has reduced on average. In 2014 its concentration among the poor has increased; however, when controlled for need, this difference is not significant.

The concentration index for curative visits was positive, indicating a pro-rich bias; a significant decline in 2014 implies that lower income groups have proportionally made more use of curative visits compared to 2006. Hence, we find that inequality in health care utilization measured by use of curative visits has significantly reduced between 2006 and 2014.

The use of a public facility for a doctor visit is more concentrated among the poor with a slight pro-rich change in 2014, changing from – 0.102 to − 0.084. This provides evidence of people in higher income quintiles increasing the use of public health care facilities and, consequently, a more equitable distribution.

Overall, these results show that inequality in health care utilization measured as utilization of curative visits is significantly reduced, a 65% change in the value of the horizontal inequity index. While the use of a public facility for a doctor visit is concentrated among the poor, the findings also confirm a reduction in socioeconomic inequality. In the next section, the Concentration Index is decomposed in different factors that account for the health care utilization measures obtained here.

### Decomposition of the concentration index

In this section, we present the results from the decomposition analysis to explain inequality in health care utilization, that is, to calculate the contribution of each variable in the analysis and its evolution in recent years. The concentration index is decomposed in different factors, computed as the product of the health variable elasticity with respect to each determinant and the concentration index of the determinant. Detailed results of the decomposition are presented in Additional file 1: Tables S1 and S2.

**Fig. 1:**
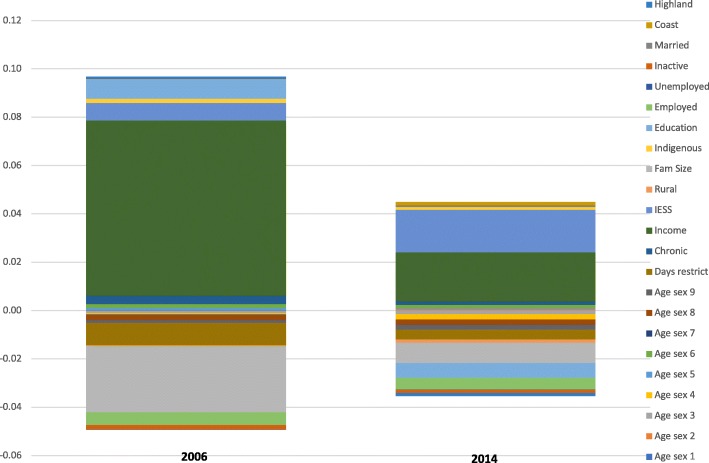
Absolute contributions to inequality in curative visit. Curative visit variable decomposition is computed considering as determinants, need and non-need variables in Table 2

Figure 1 below illustrates the compared main contributors to inequality in the curative visit variable, for years 2006 and 2014.

In Fig. 1, the absolute contributions to the concentration index (CI) of curative visits are shown. As noted before, there is remarkable pro-poor change explained mostly by the reduction in the contributions of income and family size. Specifically, the utilization of curative visits is significantly less associated with income in 2014. As one can note from Additional file 1: Table S1, income contribution declined mostly due to a reduction in the elasticity of the outcome variable with respect to income, from 0.865 to 0.234.

The contribution of family size shows an important pro-rich change, from − 0.027 to − 0.008, explained mostly by a less negative elasticity of curative visits with respect to family size. The absolute contribution of IESS affiliation also moves in a pro-rich direction in 2014, due to the increase in affiliation, in the elasticity of curative visits with respect to affiliation and in the concentration index of IESS affiliation itself. The contribution of education is also important, and it changes in a pro-poor direction, as education was positively associated to curative visits in 2006, but the sign of this association is reverted in 2014.

**Fig. 2:**
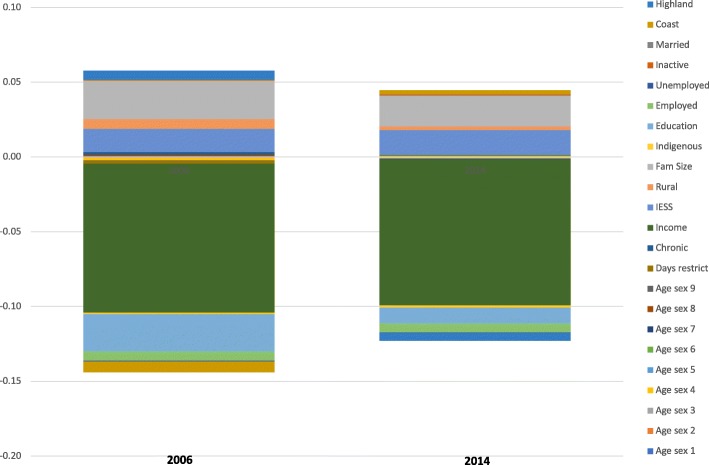
Absolute contributions to inequality in public facility for doctor visit. Public facility for the visit variable decomposition is computed considering as determinants, need and non-need variables in Table 2

For the CI of the variable public facility for the visit, that moved in a pro-rich direction from 2006 to 2014, the contribution of income was high and stable as shown in Fig. 2. The contribution of education to inequality also moved in a pro-rich direction in 2014 but still accounts for some of the inequality; this change may be explained by the negative association of years of schooling with use of public doctor visits that is not as strong in 2014 as it was in 2006. That is, better educated population is proportionally using more public doctor visits in 2014 compared to the pre-reform period.

## Discussion

This research adds to the literature on health inequality in Latin America and to the recent efforts of the Pan American Health Organization to use a standardized methodology to compare socioeconomic health inequalities across different countries in the EquiLAC II Project.

The first goal of this research is to understand the evolution of socioeconomic health inequalities in these health status and health care utilization variables. In Ecuador, health outcomes (measured by the occurrence of an illness) seem to have improved between 2006 and 2014. Also, there was an increase in utilization of curative visits, and more specifically of public facilities for doctor visits of population in all income levels. Overall, we find that inequality has significantly reduced for the utilization of curative visits, and that the use of public facilities has experienced a moderate pro-rich change. These changes might be explained by the government efforts to increase public hospital infrastructure and services and by the additional demand created by the enforcement of Constitutional Mandate 8 and the extension of health services to IESS affiliates’ children 18 and under.

Regarding our second goal, the assessment of the main determinants of inequality in health care utilization, we find that income, family size and education play a key role in the changes experienced between 2006 and 2014. The concentration index of health care utilization variables decomposition shows various results. On one side, a major reduction in the contribution of income to explaining health inequality measured by curative visits is noteworthy. On the other side, an insignificant change is observed in this contribution for public doctor visits; hence income remains as the main contributor to inequality in this case.

This paper broadens and complements Lasprilla et al. [9], contrasting with their results on the patterns of utilization of health services that benefit the population in higher income groups, and the bias towards them regarding equity. We use more recent data to assess complementary issues and find that whereas health equity has improved, there are still social and economic determinants of health such as schooling and income that require attention from policy makers.

The understanding of changes in the contribution of education to health inequality varies for each outcome. This research indicates that people with lower levels of schooling are more likely to use curative visits after the reform, as the sign of the elasticity is reversed. In the literature, see for instance Shaikh and Hatcher [29], the relationship between healthcare utilization and education is generally positive: people with higher levels of education, portray greater utilization of healthcare services, since education increases awareness about health and healthcare services use and availability. A less negative contribution of education to public doctor visits is explained by people with higher levels of schooling using more public doctor visits in 2014 than in 2006. In the case of Ecuador, the observed associations could be due to the promotion that the government applied during the reform years, such as advertising campaigns to create awareness on the right to health of the population, particularly the poor, and the increased availability of healthcare services supported by new investment in public hospitals.

IESS affiliation also matters in the explanation of health inequalities. In particular, we find a substantial increase in the absolute contribution of IESS affiliation to inequality of curative visits (from 0.007 to 0.017). This is due to the change in the CI of IESS affiliation, that moves in a pro-rich direction, and by the increase in elasticity of the outcome variable, that signals higher utilization of affiliates. As IESS affiliation was enforced, the proportion of affiliates increased from 18 to 39%; however, the undesirable effect of this policy is a change in the composition of the IESS population that seems to increase inequality.

The change in the contribution of family size to the use of a curative visit is also noticeable. Along with the reduction of 10% on the average family size from 2006 to 2014, previous to the reforms, it is possible that having a large family made it harder to attend a curative visit. Hence, the observed pattern can be explained by the inclusion of children (free of charge) and spouse (low cost) of IESS affiliates. Policies that ensure coverage, whereas generating pressure on the supply of healthcare services, might contribute to health inequality reduction.

Some findings of this study agree with those of the EquiLAC II Project. Regarding the association between health status and low-income level the findings are similar to those for Jamaica [30]. Additionally, for this case the key factors that contributed to health inequality were rural residence, unemployment and health insurance, different from the determinants in the case of Ecuador, which were income and education.

Finally, we need to mention several limitations to this study’s approach. First, there were some data constraints usually related to household surveys. The income variable is measured with error in Living Standard Measurement Surveys, although its quality has improved over time. Also, the availability of health measures in the survey constrained the number of variables we could include in the analysis. Second, the use of the golden rule for computation of the Concentration Index of binary variables has been a matter of academic debate and some alternative approaches to face the limitations are arising [27]. This is a valid concern, especially when applied to cross country analysis, which was not the case of this study, but comparability among EquiLAC II countries is constrained. Also, for binary dependent variables, non-linear models are highly recommended, yet we favor the use of a linear approximation given the feasibility to develop the decomposition analysis. Third, the results presented here cannot be understood as causal analysis. The use of a reduced form equation to estimate the contributions to CI of the health care utilization variables, might be a source of potential endogeneity, biasing the results.

## Conclusions

This is the first study that assesses the inequality implications of a public health system reform, targeted at guaranteeing the right to health, equity and social protection of the Ecuadorian population. The results show substantial changes in perceived health and healthcare utilization between 2006 and 2014. In general, the switch to a provision model that reinforces the use of public health care services, contributed to better health and a higher coverage of these services for the Ecuadorian population.

Overall, health inequalities have decreased, parallel to the reform, that shifted the model towards government provision, regulation and funding. These changes required a comprehensive set of interventions and investments to attack health and inequality determinants. The significant reduction in income contribution to inequalities in curative visits might be associated to the policy of continued increase in minimum wages. At the same time, the substantial increase in IESS affiliation contribution to inequalities in curative visits is a call for attention to its coverage policy that might be in conflict with equity goals.

These findings add to the need to better understand the evolution of health indicators and their socioeconomic determinants in Ecuador and contribute to the policy makers as feedback to focus and continue their efforts to address health inequalities. As income and education are key contributors to inequality in the utilization of health care services, it is possible to continue working in policies that affect the distribution of these variables, to this end.

## Additional file

Additional file 1: Decomposition analysis for health care utilization variables. **Table S1.** Decomposition analysis for curative visit. **Table S2.** Decomposition analysis for public facility use. (DOCX 27 kb)

## Abbreviations

CI: Concentration index
GDP: Gross domestic product
HI: Horizontal inequity index
IESS: Instituto Ecuatoriano de Seguridad Social
INEC: Instituto Nacional de Estadisticas y Censos
ISSFA: Instituto de Seguridad Social de las Fuerzas Armadas
ISSPOL: Instituto de Seguridad Social de la Policia
LSMS: Living Standards Measurement Survey
MAIS-FCI: Modelo de Atencion Integral de Salud – Familiar, Comunitaria e Intercultural
MPH: Ministry of Public Health (in Spanish, Ministerio de Salud Publica MSP)
PAHO: Pan American Health Organization
WHO: World Health Organization
