# Supplementary material for: The evolution of socioeconomic health inequalities in Ecuador during a public health system reform (2006–2014)
Source: Int J Equity Health. 2019 Feb 8;18:31. doi: 10.1186/s12939-018-0905-y (PMC6368770; doi:10.1186/s12939-018-0905-y)
Supplement: Supplementary file 1 — Decomposition analysis for health care utilization variables. Table S1. Decomposition analysis for curative visit. Table S2. Decomposition analysis for public facility use. (DOCX 27 kb) [file 12939_2018_905_MOESM1_ESM.docx]

**Additional File 1. Decomposition Analysis for health care utilization variables**

**Table S1. Decomposition Analysis for Curative Visit**

|  | **2006** | | | | **2014** | | | |
| --- | --- | --- | --- | --- | --- | --- | --- | --- |
|  | **Elasticity** | **CI_k_** | **Contrib.** | **% Contrib.** | **Elasticity** | **CI_k_** | **Contrib.** | **% Contrib.** |
| **Age sex 1** | -0.005 | 0.044 | 0.000 | -0.004 | 0.002 | 0.049 | 0.000 | 0.008 |
| **Age sex 2** | -0.006 | 0.013 | 0.000 | -0.001 | 0.016 | 0.031 | 0.001 | 0.034 |
| **Age sex 3** | 0.004 | -0.199 | -0.001 | -0.014 | 0.009 | -0.171 | -0.002 | -0.103 |
| **Age sex 4** | 0.001 | -0.359 | 0.000 | -0.008 | 0.007 | -0.314 | -0.002 | -0.147 |
| **Age sex 5** | 0.053 | 0.021 | 0.001 | 0.019 | 0.022 | 0.018 | 0.000 | 0.027 |
| **Age sex 6** | 0.019 | 0.084 | 0.002 | 0.028 | 0.022 | 0.061 | 0.001 | 0.089 |
| **Age sex 7** | 0.021 | -0.014 | 0.000 | -0.005 | 0.034 | 0.003 | 0.000 | 0.006 |
| **Age sex 8** | 0.009 | -0.233 | -0.002 | -0.036 | 0.011 | -0.210 | -0.002 | -0.153 |
| **Age sex 9** | 0.004 | -0.263 | -0.001 | -0.020 | 0.007 | -0.278 | -0.002 | -0.128 |
| **Days restrict** | 0.185 | -0.049 | -0.009 | -0.157 | 0.027 | -0.150 | -0.004 | -0.275 |
| **Chronic** | -0.068 | -0.053 | 0.004 | 0.061 | -0.042 | -0.035 | 0.001 | 0.098 |
| **Income** | 0.865 | 0.084 | 0.072 | 1.240 | 0.234 | 0.087 | 0.020 | 1.362 |
| **IESS** | 0.055 | 0.132 | 0.007 | 0.124 | 0.084 | 0.206 | 0.017 | 1.169 |
| **Rural** | 0.001 | -0.258 | 0.000 | -0.006 | 0.004 | -0.295 | -0.001 | -0.086 |
| **Fam Size** | -0.266 | 0.103 | -0.027 | -0.471 | -0.094 | 0.090 | -0.008 | -0.568 |
| **Indigenous** | -0.007 | -0.279 | 0.002 | 0.032 | -0.004 | -0.321 | 0.001 | 0.083 |
| **Education** | 0.056 | 0.146 | 0.008 | 0.140 | -0.043 | 0.141 | -0.006 | -0.408 |
| **Employed** | -0.046 | 0.114 | -0.005 | -0.090 | -0.030 | 0.161 | -0.005 | -0.327 |
| **Unemployed** | -0.002 | -0.115 | 0.000 | 0.004 | 0.001 | -0.054 | 0.000 | -0.005 |
| **Inactive** | 0.031 | -0.058 | -0.002 | -0.031 | 0.030 | -0.047 | -0.001 | -0.094 |
| **Married** | 0.010 | -0.005 | 0.000 | -0.001 | 0.038 | 0.017 | 0.001 | 0.043 |
| **Coast** | 0.001 | 0.046 | 0.000 | 0.001 | -0.040 | -0.035 | 0.001 | 0.095 |
| **Highland** | -0.017 | -0.032 | 0.001 | 0.010 | -0.024 | 0.049 | -0.001 | -0.079 |

**Table S2. Decomposition Analysis for Public Facility Use**

|  | **2006** | | | | **2014** | | | |
| --- | --- | --- | --- | --- | --- | --- | --- | --- |
|  | **Elasticity** | **CI_k_** | **Contrib.** | **% Contrib.** | **Elasticity** | **CI_k_** | **Contrib.** | **% Contrib.** |
| **Age sex 1** | -0.006 | 0.044 | 0.000 | 0.002 | 0.002 | 0.049 | 0.000 | -0.001 |
| **Age sex 2** | -0.003 | 0.013 | 0.000 | 0.000 | 0.002 | 0.031 | 0.000 | -0.001 |
| **Age sex 3** | 0.003 | -0.199 | -0.001 | 0.006 | 0.002 | -0.171 | 0.000 | 0.004 |
| **Age sex 4** | 0.004 | -0.359 | -0.001 | 0.013 | 0.002 | -0.314 | -0.001 | 0.007 |
| **Age sex 5** | 0.001 | 0.021 | 0.000 | 0.000 | 0.019 | 0.018 | 0.000 | -0.004 |
| **Age sex 6** | -0.003 | 0.084 | 0.000 | 0.003 | 0.008 | 0.061 | 0.000 | -0.006 |
| **Age sex 7** | -0.006 | -0.014 | 0.000 | -0.001 | 0.004 | 0.003 | 0.000 | 0.000 |
| **Age sex 8** | -0.006 | -0.233 | 0.001 | -0.014 | 0.000 | -0.210 | 0.000 | 0.001 |
| **Age sex 9** | 0.001 | -0.263 | 0.000 | 0.001 | -0.001 | -0.278 | 0.000 | -0.004 |
| **Days restrict** | 0.039 | -0.049 | -0.002 | 0.019 | 0.000 | -0.150 | 0.000 | -0.001 |
| **Chronic** | -0.034 | -0.053 | 0.002 | -0.017 | 0.031 | -0.035 | -0.001 | 0.013 |
| **Income** | -1.192 | 0.084 | -0.100 | 0.980 | -1.120 | 0.087 | -0.097 | 1.156 |
| **IESS** | 0.118 | 0.132 | 0.016 | -0.153 | 0.081 | 0.206 | 0.017 | -0.199 |
| **Rural** | -0.026 | -0.258 | 0.007 | -0.066 | -0.008 | -0.295 | 0.002 | -0.027 |
| **Fam Size** | 0.248 | 0.103 | 0.026 | -0.252 | 0.229 | 0.090 | 0.021 | -0.246 |
| **Indigenous** | 0.003 | -0.279 | -0.001 | 0.008 | 0.005 | -0.321 | -0.001 | 0.017 |
| **Education** | -0.171 | 0.146 | -0.025 | 0.245 | -0.073 | 0.141 | -0.010 | 0.123 |
| **Employed** | -0.056 | 0.114 | -0.006 | 0.063 | -0.038 | 0.161 | -0.006 | 0.072 |
| **Unemployed** | 0.003 | -0.115 | 0.000 | 0.004 | 0.001 | -0.054 | 0.000 | 0.001 |
| **Inactive** | -0.012 | -0.058 | 0.001 | -0.007 | -0.012 | -0.047 | 0.001 | -0.007 |
| **Married** | 0.017 | -0.005 | 0.000 | 0.001 | 0.008 | 0.017 | 0.000 | -0.002 |
| **Coast** | -0.151 | 0.046 | -0.007 | 0.069 | -0.080 | -0.035 | 0.003 | -0.033 |
| **Highland** | -0.178 | -0.032 | 0.006 | -0.057 | -0.111 | 0.049 | -0.005 | 0.064 |
